# Crocetin Attenuates Sepsis-Induced Cardiac Dysfunction *via* Regulation of Inflammatory Response and Mitochondrial Function

**DOI:** 10.3389/fphys.2020.00514

**Published:** 2020-06-09

**Authors:** Yanpeng Wang, Weiwei Yu, Chenhui Shi, Pengfei Hu

**Affiliations:** ^1^Department of Emergency, Zhejiang Chinese Medicine and Western Medicine Integrated Hospital/Hangzhou Red Cross Hospital, Hangzhou, China; ^2^Huzhou Central Hospital, Affiliated Central Hospital of Huzhou University, Hangzhou, China; ^3^The Second Clinical Medicine College of Zhejiang Chinese Medical University, Hangzhou, China; ^4^Department of Cardiology, The Second Affiliated Hospital of Zhejiang Chinese Medical University, Hangzhou, China

**Keywords:** cardiac sepsis, crocetin, cytotoxicity, inflammation, mitochondrial function

## Abstract

Sepsis-induced systemic inflammation can induce cardiac dysfunction, which can result in heart failure and death. Recently, natural drugs/compounds have received increased attention as therapeutic agents to prevent sepsis-induced cardiac dysfunction. Crocetin (CRO) is a natural compound that has been shown to reduce inflammation and cytotoxicity in cardiac ischemia/reperfusion injury. However, the effects of CRO on sepsis-induced cardiac dysfunction have not been evaluated. In this study, we used lipopolysaccharide (LPS)-induced H9c2 cells as an *in vitro* model to mimic cardiac sepsis. Crocetin significantly alleviated LPS-induced cytotoxicity, cellular apoptosis, and oxidative stress through increased Bcl-2 activity and PI3K-Akt signaling and suppression of caspase 3 and caspase 9 activities. Furthermore, CRO dramatically decreased the mRNA levels of TNF-α, IL-1, IL-6, and IL-8 via suppression of p65/Keap1 signaling and activation of Nrf2/HO-1/NQO1 signaling. In addition, CRO protected mitochondrial respiration, free fatty acid β-oxidation, and mitochondrial morphology in LPS-induced H9c2 cells. This study showed that CRO attenuated LPS-induced cardiac dysfunction via regulation of the inflammatory response and mitochondrial function and potentially had an effect on sepsis-induced cardiac dysfunction.

## Introduction

Sepsis is a systemic infection in organs that is spread in blood and is hence often described as “blood poisoning.” The host response to sepsis is characterized by proinflammatory and anti-inflammatory responses (Angus and van der Poll, 2013). Transcriptomic analysis using qPCR showed that three inflammatory markers were upregulated and four inflammatory markers were downregulated in sepsis, which indicated that systemic inflammation was associated with sepsis (Bauer et al., 2016). Myocardial depression is a well-characterized manifestation of organ dysfunction and severe inflammation in sepsis and can contribute to septic death (Rudiger and Singer, 2007; Essandoh et al., 2015). Several important inflammatory genes and factors have been shown to participate in sepsis, particularly in cardiac sepsis. The nucleotide-binding oligomerization domain-like receptor with pyrin domain (NLRP3) inflammasome and IL-1β have been shown to be expressed at high levels in a cecal ligation and puncture (CLP) sepsis model. Knockout of NLRP3 significantly reduced the plasma levels of IL-1β following CLP (Kalbitz et al., 2016). Treatment with an IκB kinase inhibitor attenuated sepsis-induced cardiac dysfunction with chronic kidney disease (CKD) via reduced expression of inflammatory cytokines (TNF-α, IL-1β, IL-6, and IL-10) (Chen et al., 2017).

Organ failure in sepsis has been associated with mitochondrial dysfunction, which contributes to impaired energy metabolism (Angus and van der Poll, 2013; Arulkumaran et al., 2016). The heart consumes a large amount of energy and is enriched in mitochondria, which produce adenosine triphosphate (ATP) and contribute to cardiac cell health and survival. Sepsis-induced cardiac dysfunction involves both the inflammatory response and mitochondrial function (Stanzani et al., 2019). Loss of PINK1 or Parkin, two mitophagy genes, increased mitochondrial stress and inhibited mitochondrial quality control, which resulted in mitochondrial DNA mutation and subsequent induction of the inflammatory response (Stolz and Dikic, 2018). Another study showed that SIRT1 and SIRT3, two proteins in the sirtuin family, regulated mitochondrial biogenesis to adapt to sepsis-induced inflammation (Liu et al., 2015). In sepsis-induced brain dysfunction, reduced tyrosine phosphorylation of the mitochondrial oxidative phosphorylation complex was observed (Lyu et al., 2015). Furthermore, septic mice exhibited severe impairment of electron transport chain (ETC) function and respiration capacity in myocardial fibers (Doerrier et al., 2016). These reports showed that sepsis induced severe mitochondrial dysfunction, and treatment using natural compounds might be a promising approach to prevent sepsis-induced cardiac dysfunction.

Several compounds have been shown to protect against septic injury. Bakuchiol (Bak) attenuated inflammation and oxidative stress in lung tissues following sepsis (Zhang et al., 2017a). Curcumin, a hydrophobic polyphenol, decreased the levels of inflammatory cytokines and protected against liver injury through regulation of PI3K/Akt, p38/JNK, and CYP2/Nrf2/ROS signaling (Zhong et al., 2016). Furthermore, another study showed that melatonin attenuated sepsis-induced cardiac dysfunction in CLP-induced rats through modulation of the PI3K/Akt pathway (An et al., 2016a). In addition, the AMPK and SIRT1 activator resveratrol (RSV) reduced apoptosis and protected cardiac function in CLP-induced cardiac sepsis through regulation of the SIRT1/FOXO1 signaling pathway (An et al., 2016b).

Crocetin (CRO) is a natural compound that has been shown to exert antioxidant effects in the heart and the brain. Mechanistic studies showed that CRO reduced cytotoxicity, apoptosis, and inflammation-related signaling in cardiac ischemia/reperfusion injury (Ishizuka et al., 2013; Huang et al., 2016; Zhang et al., 2017b). In a model of vascular dementia, CRO reversed spatial learning dysfunction and alleviated hippocampal injury (Tashakori-Sabzevar et al., 2013). Furthermore, CRO prevented disruption of mitochondrial structure and function in lung injury, which suggested that CRO promoted mitochondrial biogenesis (Venkatraman et al., 2008). Moreover, CRO has been shown to protect against cardiac ischemia/reperfusion injury through Nrf2/HO-1 signaling and the unfolded protein response pathway (revision). In this study, we hypothesized that CRO could attenuate sepsis-induced cardiac dysfunction through regulation of the inflammatory response and mitochondrial function.

## Materials and Methods

### Drugs and Chemicals

Crocetin (P0352, purity ≥ 95%) was purchased from Shanghai PureOne Biotechnology. Crocetin was dissolved in DMSO (50 mM) for storage then diluted in Dulbecco’s Modified Eagle’s Medium (DMEM) and normal saline prior to use. All chemical reagents used in this study were of analytical grade.

### Cell Culture and LPS Induction

H9c2 cardiomyocyte cells were purchased from the American Type Culture Collection (CRL1446, ATCC, United StatesA) and cultured in DMEM supplemented with 10% fetal bovine serum (FBS, Gibco, Oklahoma, OK, United States) in a humidified incubator (5% CO_2_) at 37°C. Cells were incubated with lipopolysaccharide (LPS, 1 μg/ml, Invitrogen, United States) for 6 h to induce the inflammatory response as a model of cardiac sepsis. After that, samples were collected for the subsequent analysis.

### 3-(4,5-Dimethylthiazol-2-yl)-2,5-Diphenyltetrazolium Bromide (MTT) Assay

H9c2 cell viability was determined using the MTT assay (Sigma-Aldrich, St. Louis, MO, United States). Following induction using LPS, culture medium containing MTT (500 μg/ml) was added to the plates (100 μl/well) and the cells were incubated for 3 h at 37°C. Then, DMSO was added to solubilize formazan crystals formed in viable cells. Absorbance was measured at 570 nm using a Multi-Mode plate reader (SpectraMax Paradigm, Molecular Devices, United States). Cell viability of the untreated group was set to 1.0.

### CK, LDH, SOD, MDA, and GSH-PX

Contents of superoxide Dismutase (SOD), creatine kinase (CK), lactate dehydrogenase (LDH), malondialdehyde (MDA), and glutathione peroxidase (GSH-PX) were measured in culture medium using a commercial kit (Nanjing, China) according to the manufacturer’s instructions. Following induction using LPS, the cell culture medium was collected for determination of SOD, CK, LDH, MDA, and GSH-PX content. The control group was set to a value of 1.0.

### Apoptosis

Cells were washed with PBS once and then digested using trypsin. The cells were then resuspended in PBS containing fluorescein isothiocyanate (FITC) coupled to annexin V and propidium iodide (PI; Invitrogen, United States) at room temperature in the dark for 15 min. The samples were analyzed using a BD FACSAria III flow cytometer (BD, United States) equipped with a 488-nm laser coupled to a cell sorter. Apoptotic cells were characterized by high annexin V binding and high PI staining.

### Reactive Oxygen Species and Calcium (Ca^2+^) Levels

Cellular reactive oxygen species (ROS) were quantified using the DCFDA fluorescent probe (Invitrogen, United States) according to the manufacturer’s instructions. Cellular Ca^2+^ overloading was detected using Fluo-4 (Invitrogen, United States) fluorescent probe. Following induction using LPS, cells were washed with PBS once and digested using trypsin-EDTA. The cells were resuspended in medium containing DCFDA or Fluo-4 and incubated for 30 min at 37°C in the dark. Reactive oxygen species production and calcium levels were detected using a BD FACSAria III flow cytometer (BD, United States) by monitoring the intensity of green fluorescence (Ex 485 nm/Em 535 nm). The relative intensity of the control group (DCFDA and Fluo-4) was set to 1.0.

### Reverse Transcription-Polymerase Chain Reaction

H9c2 cells were lysed and homogenized in 400 μl of lysis buffer (RLT Buffer; Qiagen, Basel, Switzerland). Then, RNA was reverse transcribed in a total volume of 20 μl using an RNA-to-cDNA Kit (Applied Biosystems, Rotkreuz, Switzerland). Quantitative RT-PCR was performed with FastStart Universal SYBR-Green Master Rox (Roche Diagnostics, Indianapolis, IN, United States) using a real-time PCR system. Primer sequences against TNF-α, IL-1, IL-6, IL-8, and β-actin (endogenous control) were used for quantitation of mRNA (primer sequences are listed in Table 1).

**TABLE 1:** Primer sequences of IL-1α, IL-1β, IL-6, IL-8, TNF-α and β-Actin.

Name	Forward	Reverse
IL-1β	5′-GCACAGTTCCCCAACTGGTA-3′	5′-AAGACACGGGTTCCATGGTG-3′
IL-6	5′-CCACCCACAACAGACCAGTA-3′	5′-GGAACTCCAGAAGACCAGAGC-3′
IL-8	5′-CTGCGCCAACACAGAAATTA-3′	5′-ATTGCATCTGGCAACCCTAC-3′
TNF-α	5′-CAGAGGGAAGAGTTCCCCAG-3′	5′-CCTTGGTCTGGTAGGAGACG-3′
β-Actin	5′-GCTACAGCTTCACCACCACA-3′	5′-ATCGTACTCCTGCTTGCTGA-3′

### Oxygen Consumption Rate

Mitochondrial oxygen consumption rate (OCR) was measured by the XF Cell Mito Stress Test using a Seahorse XFp Extracellular Flux Analyzer (Seahorse Bioscience, United States). H9c2 cells were seeded on XFp cell culture miniplates 48 h prior to LPS induction and CRO treatment. Following CRO treatment, ports A, B, and C on the sensor cartridge were injected with 1.5 μM oligomycin (inhibitor of complex V), 2 μM FCCP, and 0.5 μM rotenone/antimycin A (inhibitors of complex I and complex III), respectively. Following sensor calibration, the sensor was placed on the cell plate and the mitochondrial stress test was performed. Mitochondrial OCR was calculated according to standardized procedures and normalized to protein concentrations in each well.

### Adenosine Triphosphate and β-Hydroxybutyrate Measurement

Adenosine triphosphate production was measured by using a Luminescent ATP Detection Assay Kit (Abcam, United Kingdom). Levels of ATP were determined in three independent experiments and were measured using a Multi-Mode detection platform.

Levels of β-hydroxybutyrate (BHB) were determined using a colorimetric assay kit (Cayman Chemical Company, United States). Cells (∼18 × 10^6^ cells) were collected using a rubber policeman and centrifuged at 2,000 *g* for 10 min at 4°C. Cell pellets were resuspended in 1–2 ml of cold assay buffer and sonicated 20 times (in 1-s bursts) and then centrifuged at 10,000 *g* for 10 min. The supernatants were removed, and the pellets were resuspended in 1 ml of cold assay buffer and stored on ice. Each sample was prepared for quantitation by combining 50 μl of sample with 50 μl of developer solution. The plates were incubated at 25°C in the dark for 30 min and then analyzed at 445–455 nm. The relative absorbance of the control group was set to 1.0.

### Mitochondrial Morphology

H9c2 cells were seeded onto coverslips in clear-bottom confocal dishes at a density of 50,000 cells per dish. Following treatment with LPS, the cells were incubated with 30 nM MitoView Red (GeneCopoeia, United States) at 37°C for 30 min. The cells were then washed with PBS three times. Mitochondrial morphology was visualized using a confocal microscope equipped with a 60× oil immersion objective. Red fluorescence represented the mitochondria, and the lengths of the mitochondria were measured.

### Immunoblot Analysis

H9c2 cells were lysed in ice-cold RIPA buffer (Cell Signaling Technology, United States) containing protease inhibitor, then incubated on ice for 30 min and centrifuged at 13,000 *g* for 15 min at 4°C. The supernatants were collected, and protein content was measured. Proteins (30 μg/lane) were loaded and separated by 10% SDS-PAGE and then transferred to PVDF membranes (pore size: 0.45 μm, Millipore). The membranes were blocked using 5% non-fat milk in TBST (Tris-buffered saline with 0.05% Tween 20) for 1 h, then incubated with primary antibodies against cytochrome c (1:1,000), Bcl-2 (1:500), Bax (1:1,000), caspase 3 (1:500), caspase 9 (1:500), PI3K (1:1,000), p-PI3K (1:1,000), p-Akt (1:1,000), Akt (1:1,000), p-p65 (1:1,000), p65 (1:1,000), NQO1 (1:1,000), Keap1 (1:1,000), Nrf2 (1:1,000), HO-1 (1:1,000), lamin B (1:1,000), ACAT1 (1:2,000), OXCT1 (1:2,000), Mfn1 (1:1,000), Mfn2 (1:2,000), OPA1 (1:1,000), Drp1 (1:2,000), Fis1 (1:2,000), MFF (1:1,000), Mid49 (1:1,000), and Mid51 (1:1,000, Cell Signaling Technology, United States) overnight at 4°C. Beta-Actin (1:10,000, Sigma, United States) was used as the loading control. Following incubation with primary antibodies, the membranes were washed with TBST three times (5 min each) and then incubated with secondary antibodies (1:10,000) for 2 h at room temperature. The intensities of bands were determined using ECL, and images were captured using a ChemiDoc Touch Imaging System (Bio-Rad, United States).

### Statistics

All data are expressed as means ± SEM. Student’s *t*-test was used to compare two groups, and one-way analysis of variance (ANOVA) with multiple comparisons using Turkey’s test was used to analyze differences between three or more groups. *p* < 0.05 was considered statistically significant.

## Results

### Crocetin Alleviated Myocardial Toxicity in LPS-Induced H9c2 Cardiomyocytes

**FIGURE 1:**
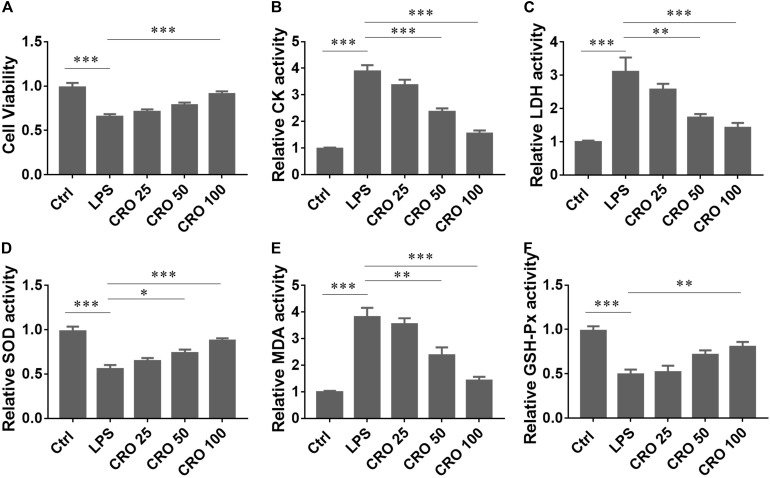
Effect of CRO on cytotoxic indices in LPS-induced cardiac sepsis. **(A)** Effect of CRO on cell viability of LPS-induced H9c2 cells. Data (*n* = 6) are presented as the mean ± SEM, ***p < 0.001. **(B,C)** Effect of CRO on CK and LDH in LPS-induced H9c2 cells. Data (*n* = 6) are presented as the mean ± SEM, **p < 0.01, ***p < 0.001. **(D–F)** Effect of CRO on SOD, MDA, and GSH-PX in LPS-induced H9c2 cells. Data (*n* = 6) are presented as the mean ± SEM, *p < 0.05, **p < 0.01, ***p < 0.001.

To determine whether CRO attenuated sepsis-induced cardiac dysfunction, we used an LPS-induced H9c2 cell *in vitro* model of sepsis (Kalbitz et al., 2016; Zhang et al., 2017c). The results from the MTT assay showed that CRO significantly increased cell survival in a dose-dependent manner in LPS-induced H9c2 cells (Figure 1A). We also measured the activities of CK and LDH, two important cardiac enzymes. The results showed that induction using LPS increased the contents of CK and LDH. Treatment with CRO reduced the activities of CK and LDH in a dose-dependent manner (Figures 1B,C). As oxidative stress plays an important role in cardiac function disorders, we analyzed whether CRO reduced oxidative stress. The results showed that CRO significantly upregulated the activities of SOD and GSH-Px and reduced the level of MDA in LPS-induced H9c2 cells (Figures 1D–F). These results showed that CRO alleviated myocardial toxicity in LPS-induced sepsis.

### Crocetin Alleviated Myocardial Apoptosis in LPS-Induced H9c2 Cardiomyocytes

**FIGURE 2:**
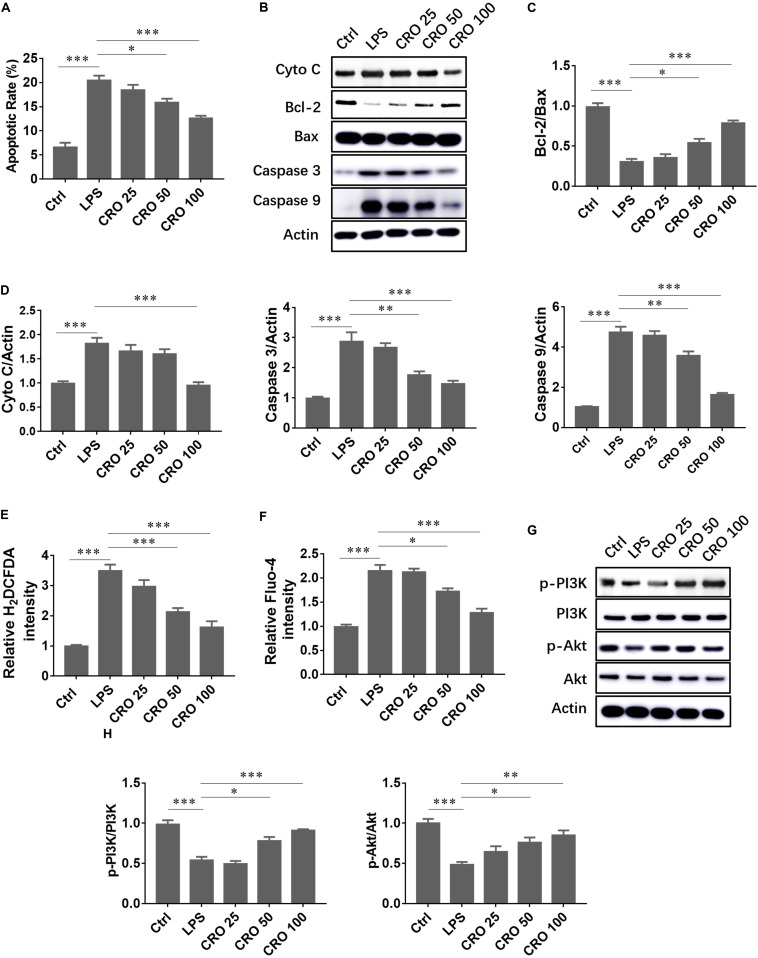
Effect of CRO on cell apoptosis and cell survival in LPS-induced cardiac sepsis. **(A)** Effect of CRO on rate of apoptosis in LPS-induced H9c2 cells. Data (*n* = 3) are presented as the mean ± SEM, *p < 0.05, ***p < 0.001. **(B)** Effect of CRO on the expression of cytochrome c, Bcl-2, Bax, caspase 3, and caspase 9 in LPS-induced H9c2 cells. The ratios of Bcl-2 to Bax and quantitation of cytochrome c, caspase 3, and caspase 9 are summarized in panels **(C,D)**, respectively. Data (*n* = 3) are presented as the mean ± SEM, *p < 0.05, **p < 0.01, ***p < 0.001. **(E,F)** Effect of CRO on ROS (indicated by H_2_DCFDA) and Ca^2+^ (using Fluo-4 as an indicator) levels as determined using flow cytometry in LPS-induced H9c2 cells. Data (*n* = 3) are presented as the mean ± SEM, *p < 0.05, ***p < 0.001. **(G)** Effect of CRO on the expression of p-PI3K, PI3K, p-Akt, and Akt in LPS-induced H9c2 cells. Levels of these proteins were quantitated and are presented in panel **(H)**. Data (*n* = 3) are presented as the mean ± SEM, *p < 0.05, ***p < 0.001.

We evaluated whether CRO suppressed LPS-induced cardiomyocyte apoptosis. The results showed that LPS significantly induced cell apoptosis, downregulated the expression of the anti-apoptosis protein Bcl-2, and upregulated the expression of caspase proteins. Crocetin reduced apoptosis and the ratio of Bcl-2 to Bax (Figures 2A–C). Moreover, immunoblot analysis of cytochrome c, caspase 3, and caspase 9 expression showed that CRO significantly attenuated LPS-induced myocardial apoptosis (Figures 2B,D). As sepsis-induced cardiac dysfunction generally results in excessive ROS production and calcium overloading, we evaluated whether CRO influenced ROS and calcium levels. Flow cytometry results showed that CRO decreased green fluorescence in a dose-dependent manner, which indicated that levels of ROS and calcium were reduced (Figures 2E,F). Moreover, CRO suppressed myocardial apoptosis and enhanced cell survival signaling. Western blot analysis showed that CRO significantly upregulated the expression of phospho-PI3K and phospho-Akt in LPS-induced septic cardiomyocytes (Figures 2G,H).

### Crocetin Alleviated LPS-Induced Sepsis by Regulating Inflammatory Response in Cardiomyocytes

**FIGURE 3:**
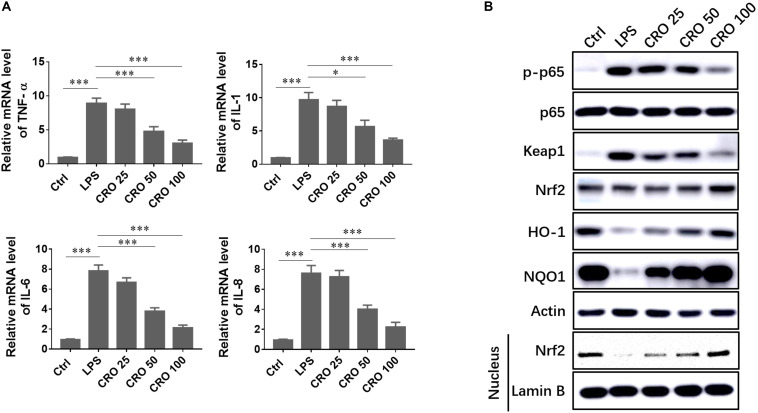
Effect of CRO on the inflammatory response in LPS-induced cardiac sepsis. **(A)** Effect of CRO on proinflammatory cytokines in LPS-induced H9c2 cells. Data (*n* = 3) are presented as the mean ± SEM, *p < 0.05, ***p < 0.001. **(B)** Effect of CRO on the expression of p-p65, p65, keap1, Nrf2, HO-1, and NQO1 and nuclear expression of Nrf2 in LPS-induced H9c2 cells.

Sepsis-induced cardiac dysfunction has been shown to induce a strong inflammatory response. We evaluated the ability of CRO to enhance cardiomyocyte survival through reduced cellular inflammation. We showed that the mRNA expression levels of classical inflammatory cytokines, such as TNF-α, IL-1, IL-6, and IL-8, were increased in response to LPS treatment. Treatment with CRO significantly reduced the expression levels of these inflammatory cytokines, which indicated that CRO could reduce sepsis-induced inflammation (Figure 3A). To further characterize the mechanisms by which CRO reduced cardiac inflammation, we evaluated relevant signaling pathways. Induction using LPS resulted in upregulation of p-p65, but not total p65 expression, and in upregulation of Keap1. Treatment with CRO resulted in dose-dependent decreases in the expression levels of p-p65 and Keap1, which indicated an anti-inflammatory effect. We then evaluated Nrf2/HO-1/NQO1 signaling, which contributes to anti-oxidant-mediated modulation of the inflammatory response. Western blot results showed that CRO significantly upregulated the expression levels of Nrf2, HO-1, and NQO1, each of which were downregulated by LPS. We also showed that Nrf2 expression in the nucleus was significantly reduced by treatment with LPS and that CRO protected against this decrease (Figure 3B).

### Crocetin Alleviated LPS-Induced Sepsis by Regulating Mitochondrial Biogenesis in Cardiomyocytes

**FIGURE 4:**
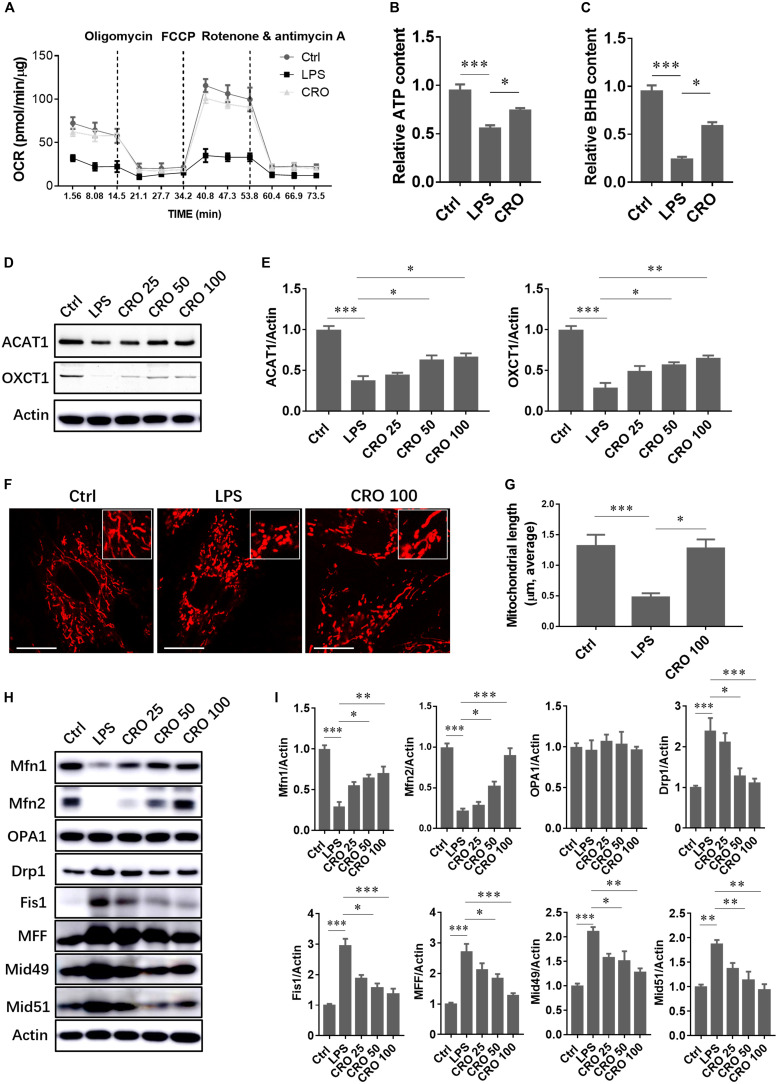
Effect of CRO on mitochondrial function and morphology in LPS-induced cardiac sepsis. **(A)** Effect of CRO on mitochondrial respiratory function in LPS-induced H9c2 cells. Data (*n* = 3) are presented as the mean ± SEM. **(B,C)** Effect of CRO on levels of ATP and BHB in LPS-induced H9c2 cells. Data (*n* = 3) are presented as the mean ± SEM. *p < 0.05, ***p < 0.001. **(D)** Effect of CRO on the expression of ACAT1 and OXCT1 in LPS-induced H9c2 cells. Expression levels are summarized in panel **(E)**. Data (*n* = 3) are presented as the mean ± SEM. *p < 0.05, **p < 0.01, ***p < 0.001. **(F)** Effect of CRO (100 μM) on mitochondrial morphology in LPS-induced H9c2 cells. Mitochondrial length was determined in 50 individual cells using Leica software **(G)**. Data (*n* = 3) are presented as the mean ± SEM, *p < 0.05, ***p < 0.001. **(H)** Effect of CRO on the expression of mitochondrial fusion and fission proteins in LPS-induced H9c2 cells. Expression levels are summarized in panel **(I)**. Data (*n* = 3) are presented as the mean ± SEM. *p < 0.05, **p < 0.01, ***p < 0.001.

Disruption of mitochondrial morphology and function is a molecular characteristic of cardiac dysfunction and is also observed in LPS-induced sepsis. The protective effects of CRO against cardiac sepsis through regulation of mitochondrial biogenesis have not been characterized. A significant reduction in mitochondrial OCR was detected in LPS-induced cardiomyocytes, which showed that LPS-induced sepsis caused disruption of mitochondrial respiration. Treatment with CRO protected against LPS-induced mitochondrial respiratory dysfunction (Figure 4A). We further analyzed the levels of the energy metabolites ATP and BHB. The results showed that treatment with CRO resulted in increased levels of ATP and BHB, which indicated that CRO improved energy metabolism in septic cardiomyocytes (Figures 4B,C). We also showed that CRO restored BHB-related fatty acid β-oxidation via upregulation of ACAT1 and OXCT1, two important enzymes involved in BHB biotransformation (Figures 4D,E).

Mitochondrial morphology changes continuously to adapt to the intracellular microenvironment, particularly in response to oxidative stress or inflammation. Mitochondrial morphology was analyzed by confocal microscopy using MitoTracker staining. Red fluorescence indicated mitochondria. Following induction using LPS, mitochondrial lengths were significantly reduced. Treatment with CRO reversed LPS-induced effects on mitochondrial morphology (Figures 4F,G). To further characterize the mechanisms by which CRO regulated mitochondrial morphology, we measured the expression levels of proteins involved in mitochondrial fusion and fission. Immunoblot analysis showed that CRO upregulated fusion proteins that were downregulated by LPS in a dose-dependent manner. In addition, CRO downregulated fission proteins that were upregulated by LPS, which suggested that CRO restored mitochondrial morphology in LPS-induced cardiomyocytes through regulation of fusion and fission (Figures 4H,I).

## Discussion

Our study showed that CRO protected against LPS-induced cardiac dysfunction in an *in vitro* cell model with H9c2 cardiomyocytes, which may have been due to inhibition of inflammation (Nam et al., 2010; Song et al., 2016) and regulation of mitochondrial function (Venkatraman et al., 2008). Previous studies showed that CRO exerted cardioprotective effects, particularly against cardiac ischemia/reperfusion injury. However, the protective effects of CRO against other cardiac injuries, such as sepsis-induced cardiac dysfunction, have not been characterized. We showed that CRO potentially prevented sepsis-induced cardiac dysfunction *via* regulation of the inflammatory response and mitochondrial function in H9c2 cardiomyocytes.

Previous studies showed that cardiac cell apoptosis occurs in *in vivo* and *in vitro* models of sepsis. Compounds that attenuate sepsis-induced cardiac dysfunction should also have the ability to decrease cell apoptosis. Phenylephrine, an alpha-1 adrenergic receptor agonist, alleviated sepsis-induced cardiac dysfunction by inhibiting apoptosis (Li et al., 2019). Caspase inhibitor, endotoxin, and the natural compound melatonin prevented cardiac apoptosis and cardiac dysfunction in a rat model of sepsis (Neviere et al., 2001; Zhang et al., 2019). In our study, CRO significantly reduced H9c2 cell apoptosis in a dose-dependent manner and upregulated the expression of the anti-apoptosis protein Bcl-2 and the ratio of Bcl-2 to Bax. Moreover, CRO reduced myocardial oxidative stress by decreasing ROS production and preventing calcium overloading. Finally, CRO upregulated cell survival signaling through the PI3K/Akt pathway, which prevented apoptosis.

We also evaluated mitochondrial oxidative phosphorylation and free fatty acid β-oxidation, which occurs in the liver. The liver transports products of energy metabolism to other organs, such as the heart. Metabolic products of β-oxidation include ketone bodies such as BHB, acetoacetate, and acetone, among which BHB is the most abundant (∼75%) (Cotter et al., 2013). Induction using LPS resulted in significantly decreased levels of BHB, and CRO prevented this decrease. To characterize the mechanisms of regulation of BHB production, we measured the expression levels of acetyl-CoA acetyltransferase 1 (ACAT1) and succinyl-CoA:3-ketoacid coenzyme A transferase (OXCT1). Succinyl-CoA:3-ketoacid coenzyme A transferase catalyzes the transfer of CoA from succinyl-CoA to acetoacetate to form acetoacetyl-CoA, and ACAT1 transfers an acetyl group to free CoA, resulting in the generation of two acetyl-CoA molecules. Each of these steps is essential for the production of ketone bodies and their transport to extrahepatic tissues (Fukao et al., 2014; Newman and Verdin, 2014). These findings showed that CRO upregulated the expression of ACAT1 and OXCT1 to promote the utilization of ketone bodies in cardiomyocytes.

Mitochondria are dynamic organelles that undergo fusion and fission to adapt to the intracellular environment. The heart consumes a large amount of energy, and healthy mitochondria are necessary to produce adequate levels of ATP. We observed mitochondrial morphology and measured the expression levels of mitochondrial fusion and fission proteins. Induction using LPS resulted in significant mitochondrial fragmentation. Treatment with LPS resulted in the downregulation of the mitochondrial fusion proteins Mfn1, Mfn2, and OPA1 and the upregulation of the mitochondrial fission proteins Drp1, Fis1, MFF, Mid49, and Mid51. Treatment with CRO prevented mitochondrial fragmentation and inhibited LPS-induced changes in expression levels of mitochondrial fusion and fission proteins.

Taken together, our study showed that CRO potentially alleviated sepsis-induced cardiac dysfunction by reducing cytotoxicity, cell apoptosis, oxidative stress, inflammation, and mitochondrial dysfunction, and by promoting maintenance of normal cardiomyocyte function.

## Data Availability Statement

The raw data supporting the conclusions of this article will be made available by the authors, without undue reservation, to any qualified researcher.

## Author Contributions

PH and YW contributed the conception and design of the study and wrote the first draft of the manuscript. YW and WY contributed acquisition, analysis, or interpretation of data for the work. CS performed the statistical analysis. PH took primary responsibility for communication with the journal and editorial office during the submission process, throughout peer review, and during publication. All authors contributed to manuscript revision and read and approved the submitted version.

## Conflict of Interest

The authors declare that the research was conducted in the absence of any commercial or financial relationships that could be construed as a potential conflict of interest.
